# Reduction of the Hawaiian genus *Platydesma* into Melicope section Pelea (Rutaceae) and notes on the monophyly of the section

**DOI:** 10.3897/phytokeys.91.21363

**Published:** 2017-12-19

**Authors:** Marc S. Appelhans, Kenneth R. Wood, Warren L. Wagner

**Affiliations:** 1 Department of Systematics, Biodiversity and Evolution of Plants Botany, Albrecht-von-Haller Institute of Plant Sciences, University of Goettingen, Untere Karspuele 2, 37073 Goettingen, Germany; 2 Department of Botany, National Museum of Natural History, Smithsonian Institution, P.O. Box 37012, MRC 166, Washington, DC 20013-7012, USA; 3 National Tropical Botanical Garden, 3530 Papalina Road, Kalāheo, HI 96741, USA

**Keywords:** Hawaiian Islands, *Melicope*, New Caledonia, *Platydesma*, Rutaceae

## Abstract

*Platydesma*, an endemic genus to the Hawaiian Islands containing four species, has long been considered of obscure origin. Recent molecular phylogenetic studies have unequivocally placed *Platydesma* within the widespread genus *Melicope* as sister to the rest of the Hawaiian species of *Melicope*. This makes submerging *Platydesma* into *Melicope* necessary. We make the necessary new combinations: *Melicope cornuta* (Hillebr.) Appelhans, K.R. Wood & W.L. Wagner, M. cornuta var. decurrens (B.C.Stone) Appelhans, K.R. Wood & W.L. Wagner, *M. remyi* (Sherff) Appelhans, K.R. Wood & W.L. Wagner, and *M. rostrata* (Hillebr.) Appelhans, K.R. Wood & W.L. Wagner. An additional species that has been recognized within *Platydesma* should now be recognized under its original name *M. spathulata* A. Gray. All Hawaiian species belong to Melicope section Pelea. Our molecular phylogenetic studies also showed that in addition to merging Platydesma into section Pelea, five species described from New Caledonia need to be excluded from the section in order to achieve monophyly of section Pelea.

## Introduction and discussion

The genus *Melicope* J.R. Forst. & G. Forst. is the largest genus within Rutaceae, with approximately 230 species ranging throughout the Malagasy and Indo-Himalayan regions, Southeast Asia, Australasia, and across the Pacific Islands (Hartley 2001). One of the centers of diversity is the Hawaiian Islands, where *Melicope* (including *Platydesma*) is the fourth largest radiation with 54 species after the Hawaiian Lobeliads (Campanulaceae), *Cyrtandra* J.R. Forst. & G. Forst. (Gesneriaceae), and mints (Lamiaceae) (Hartley and Stone 1989, Wagner et al. 1990, Hartley 2001, Wood et al. 2017). It furthermore represents the largest radiation of woody plants on the Hawaiian Islands (Wagner et al. 1990). Hawaiian *Melicope* are an example of an adaptive radiation, as the genus has undergone spectacular morphological and ecological diversification (Stone 1966, Carlquist 1974). Hawaiian *Melicope* taxa, and another rutaceous genus *Platydesma* H. Mann have been widely decimated throughout the Hawaiian Islands due to habitat alteration and introduced organisms; many extant species of *Melicope* and all taxa within *Platydesma* except for *P. spathulata* (A. Gray) B.C. Stone are considered rare, vulnerable, or endangered. At least five species of *Melicope*, namely *M. balloui* (Rock) T.G. Hartley & B.C. Stone, *M. macropus* (Hillebr.) T.G. Hartley & B.C. Stone, *M. nealae* (B.C. Stone) T.G. Hartley & B.C. Stone, *M. obovata* (H. St. John) T.G. Hartley & B.C. Stone and *M. wailauensis* (H. St. John) T.G. Hartley & B.C. Stone, are presumed to be extinct and 11 species are known from 50 or less living individuals in the wild (Wagner et al. 1999, Wood 2011, 2014, Wood et al. 2016).

The immediate relationships of the Hawaiian endemic genus *Platydesma* (Rutaceae) have puzzled taxonomists due to the divergent floral morphology and hermaphroditic breeding system of *Platydesma* (Stone 1962a, Wagner et al. 1990, Funk and Wagner 1995). Engler (1931) placed *Platydesma* between the North American (Mexico and southwestern USA) genus *Choisya* Kunth and the New Caledonian genus *Dutaillyea* Baill., while Stone (1962a) hypothesized that the genus was derived from the Australian, New Caledonian and New Guinean genus *Medicosma* Hook. f. Even though *Dutaillyea* proved to be part of *Melicope*, and *Medicosma* is the sister genus of *Melicope*, neither of these taxa are immediate relatives of *Platydesma* and the Hawaiian *Melicope* species (Appelhans et al. 2014a). While most *Melicope* species have a dimorphic breeding system (Sakai et al. 1995, Hartley 2001), *Platydesma* is a peculiar taxon for its monadelphous stamens and bisexual flowers (Hillebrand 1888, Kubitzki et al. 2011). In addition, *Platydesma* has unique chemical characteristics; the leaves, bark and wood emit a semeniferous odor due to the unique alkaloid platydesmine (Werny and Scheuer 1963).

**Figure 1.**
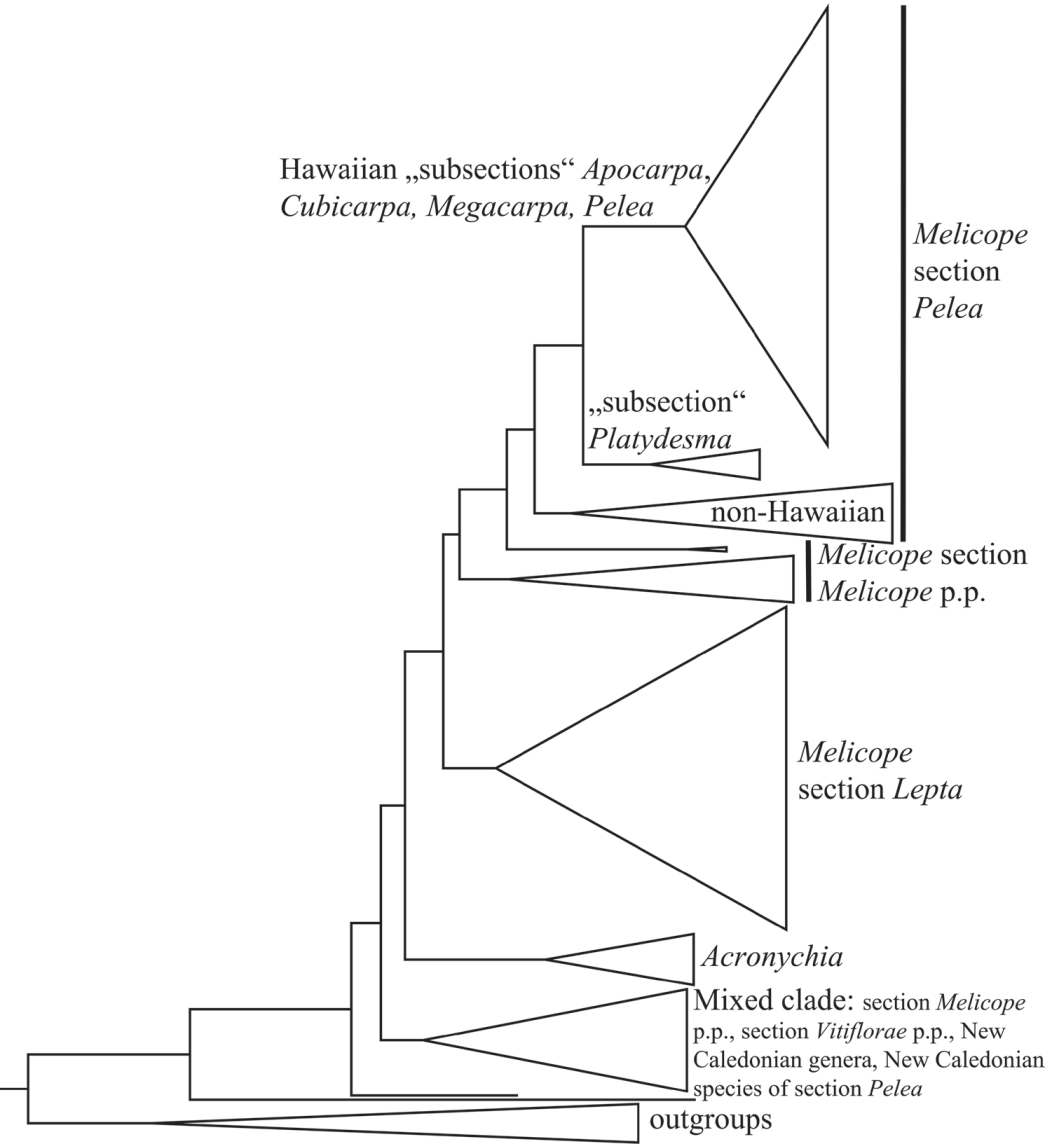
Phylogenetic affinities of Hawaiian Melicope section Pelea (incl. *Platydesma*) based on five plastid and nuclear markers (modified from Appelhans et al. 2014a). “Subsections” *Apocarpa*, *Megacarpa* and *Pelea* contain exclusively Hawaiian and Marquesan species and “subsection” *Platydesma* includes the former Hawaiian genus *Platydesma*. The New Caledonian species of Melicope section Pelea are part of the “mixed clade” and we therefore propose to exclude them from section Pelea.

Recent molecular phylogenetic studies provided unequivocal evidence for the placement of *Platydesma* within *Melicope*, as sister to the Hawaiian taxa in Melicope section Pelea (Harbaugh et al. 2009, Appelhans et al. 2014a, 2014b; Fig. 1). These data are consistent with Carlquist’s (1974) hypothesis of a Hawaiian origin of *Platydesma*, as well as with Asa Gray’s classification of *Platydesma* as *Melicope* taxa (Stone 1962b). This represents an example of divergent evolution in an insular setting. Despite the differences in morphology and breeding system, however, the seedling stages of *Melicope* and *Platydesma* are homologous (Stone 1962b).

The Hawaiian Islands have the highest rate of dioecy in the world, evolving *in situ* in at least 12 lineages, possibly as a selective force to avoid inbreeding depression, affect resource allocation, and sexual selection (Sakai et al. 1995). Sakai et al. (1995) hypothesized that Hawaiian *Melicope* and *Platydesma* both arose from separate colonizations; the *Melicope* colonist was dimorphic while the ancestor of *Platydesma* was monomorphic. The results of molecular phylogenetic analyses (Harbaugh et al. 2009, Appelhans et al. 2014a,b) demonstrate that Hawaiian *Melicope* and *Platydesma* most likely arose from a single ancestor. The ancestor of Hawaiian *Melicope* + *Platydesma* was likely dioecious, because the closest relatives of the Hawaiian species (=the remainder of Melicope section Pelea excluding species from New Caledonia) are almost exclusively dioecious (Hartley 2001). Only the widespread *M. triphylla* (Lam.) Merr. and the New Guinean endemic *M. conjugata* T.G. Hartley, which are normally dioecious, are in rare cases monoclinous (*M. triphylla*, *M. conjugata*) or andromonoecious (*M. triphylla*). Therefore, *Platydesma* represents a rare reversal from dioecy to synoecy as Carlquist (1974) had hypothesized. Despite the differences in flower, seed and fruit characteristics (*Melicope* has dehiscent fruits while *Platydesma* has indehiscent or tardily dehiscent fruits), Sakai et al. (1995) hypothesized that both genera are insect pollinated, and have undergone long distance dispersal through bird ingestion. Therefore, the mechanisms that may have lead to this reversal in breeding system are unknown, but with discovery of large quantities of nectar produced in the flowers it is likely the breeding system change is part of the shift to bird pollination.

The copious nectar production and the stamens connate into a cup-like structure that holds accumulating nectar in *Platydesma* flowers suggest adaptations to bird-pollination. A similar case of adaptation to bird-pollination can be found in Hawaiian *Schiedea*/*Alsinidendron* (Caryophyllaceae) (Weller et al. 1998, Golonka et al. 2005, Wagner et al. 2005). Like in *Melicope*/*Platydesma*, adaptation to different pollination vectors has resulted in differences in flower morphology between *Schiedea* and *Alsinidendron* that are virtually identical to those in *Melicope*/*Platydesma*, which led to the separation of the group into two genera.

To preserve the monophyly of Melicope section Pelea, *Platydesma* must be merged with *Melicope* and the New Caledonian species of section Pelea have to be excluded (Fig. 1). Hawaiian *Melicope* have been subdivided into the four groups *Apocarpa*, *Cubicarpa*, *Megacarpa* and *Pelea*, which were regarded as sections within the genus *Pelea* A. Gray (Stone 1969, Wagner et al. 1990). Now that Pelea occupies the rank of a section within Melicope, these four groups perhaps should be regarded as subsections. Due to the significant morphological differences between Hawaiian *Melicope* and *Platydesma* (Wagner et al. 1990), a new subsection would need to be created to accommodate *Platydesma.* However, since our molecular phylogenetic studies (Appelhans et al. 2014b) showed low resolution concerning some of the Hawaiian groups, it is premature to establish a new subsectional classification. Instead, we will await the results of our recently initiated Next-Generation Sequencing project focused on Hawaiian *Melicope*.

Hartley (2001) placed the five New Caledonian species of Melicope in section Pelea. He mentions that their “immediate and broader relationships within sect. Pelea are not clear” (Hartley 2001; p. 139) and that they “are probably relicts” (Hartley 2001; p. 31). Hartley listed some characters that might connect the New Caledonian and the Hawaiian species (e.g., persistent petals in three New Caledonian and two Hawaiian species, infertile antepetalous stamens in three New Caledonian species and rare occurrences in Hawaiian species). He also mentioned characters of the New Caledonian species that do not occur in other species of the section or the genus, among which are: abruptly acuminate flower buds, and persistent and accrescent sepals and/or petals (Hartley 2001; p. 140). Molecular phylogenetic studies (Appelhans et al. 2014a,b; Fig. 1) have shown that the New Caledonian species of section Pelea are not directly related to the Hawaiian species and the remainder of section Pelea, but that they belong to a clade of taxa from New Caledonia, Australia, New Zealand and the South Pacific, including *Melicope* sections *Melicope* p.p. and *Vitiflorae* (F. Muell.) T.G. Hartley p.p., as well as the genera *Comptonella* Baker f., *Dutaillyea*, *Picrella* Baill. and *Sarcomelicope* Engl. (Appelhans et al. 2014b). In line with the results of the phylogenetic reconstruction and the unusual characters mentioned by Hartley (2001), we propose to exclude the New Caledonian species from Melicope section Pelea.

## Taxonomy

**Figure 2.**
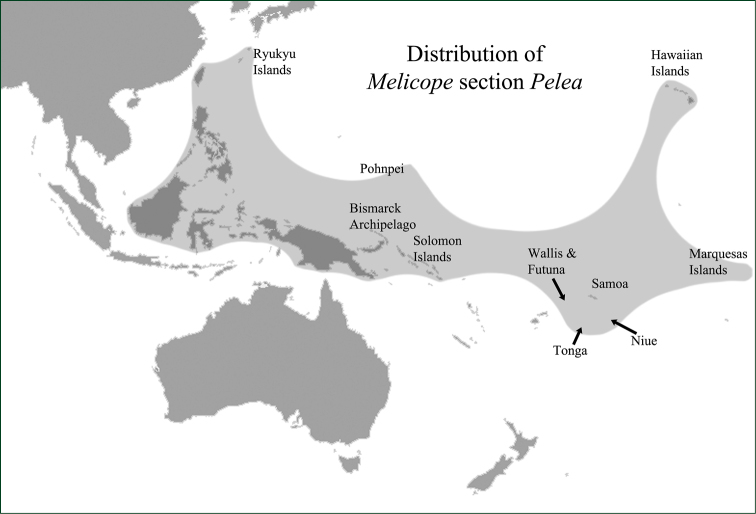
Distribution of the newly circumscribed Melicope section Pelea.

With four species of *Platydesma* included and five New Caledonian species excluded, Melicope section Pelea consists of 86 currently recognized species (Hartley 2001, Wood et al. 2017). The distribution of the section ranges from Borneo, the Philippines, Taiwan and the Ryukyu Islands to the Hawaiian and Marquesas Islands. In the Pacific, the section occurs on the Ryukyu Islands, Pohnpei (Caroline Islands), Bismarck Archipelago, Solomon Islands, Wallis and Futuna, Tonga, Samoa, Niue, Hawaiian Islands, and Marquesas Islands (Hartley 2001; Fig. 2). In the following synopsis sheet numbers, when available are cited for holotype specimens, and barcode numbers are provided for specimens in brackets [].

We propose the following taxonomic changes for *Platydesma*:

### Melicope sect. Pelea

Taxon classificationPlantaeSapindalesRutaceae

(A. Gray). Hook. f., Gen. Pl. 1: 296. 1862.

Platydesma H. Mann, *Proc. Bost. Soc. Nat. Hist.* 10: 317. 1866. – Type: Platydesma campanulata H. Mann (=Melicope spathulata A. Gray)
Platydesma sect. Cornutia B.C. Stone, *J. Arnold Arbor*. 43: 422. 1962. – Type: Platydesma cornuta Hillebr. (=Melicope cornuta (Hillebr.) Appelhans, K.R. Wood & W.L. Wagner).

#### Type.

*Pelea clusiifolia* A. Gray (=*Melicope clusiifolia* (A. Gray) T.G. Hartley & B.C. Stone).

### Melicope cornuta

Taxon classificationPlantaeSapindalesRutaceae

(Hillebr.) Appelhans, K.R. Wood & W.L. Wagner comb. nov.

urn:lsid:ipni.org:names:77174269-1

Fig. 3D

Platydesma cornuta Hillebr., *Fl. Hawaiian Isl.* 72. 1888.

#### Note.

As was typical for Hillebrand in the Flora of the Hawaiian Islands, he cited localities for which he saw collections and would only sometimes cite collector information (when someone other than himself made the collection). In this case he cited three localities (Halemano, Wailupe, and Pauoa) indicating he collected or saw material from each one of them. As such these collections must be considered syntypes. Stone (1962a) indicated the K sheet and the GH sheet cited below as holotype and isotype. They represent inadvertent selection of a lectotype and isolectotype. A number of additional syntypes from all three cited localities are known but can with certainty not be considered isolecotypes because the two specimens selected by Stone have no locality on the label other than O‘ahu and Kaua‘i.

#### Type.

O‘ahu: *s. l.*, *W. Hillebrand s.n.* (lectotype, designated by Stone, *J. Arnold Arbor.* 43: 423. 1962: K [K000717606, image!]; isolectotype: GH [GH00044164, image!]. Additional syntypes: O‘ahu, Halemanu, *Hillebrand s.n.* (BISH [BISH1016374, specimen!], US [US00101497, specimen!]); O‘ahu, Pauoa Valley, *Hillebrand & J. M. Lydgate s.n.* (BISH [2; BISH1016375, BISH1016476, specimens!]; O‘ahu, Wailupe (MEL [MEL587728, image!], with a photo at BISH); and a fragment made by J. Rock of B sheet, without locality (BISH [BISH1016377, specimen!]).

**Figure 3.**
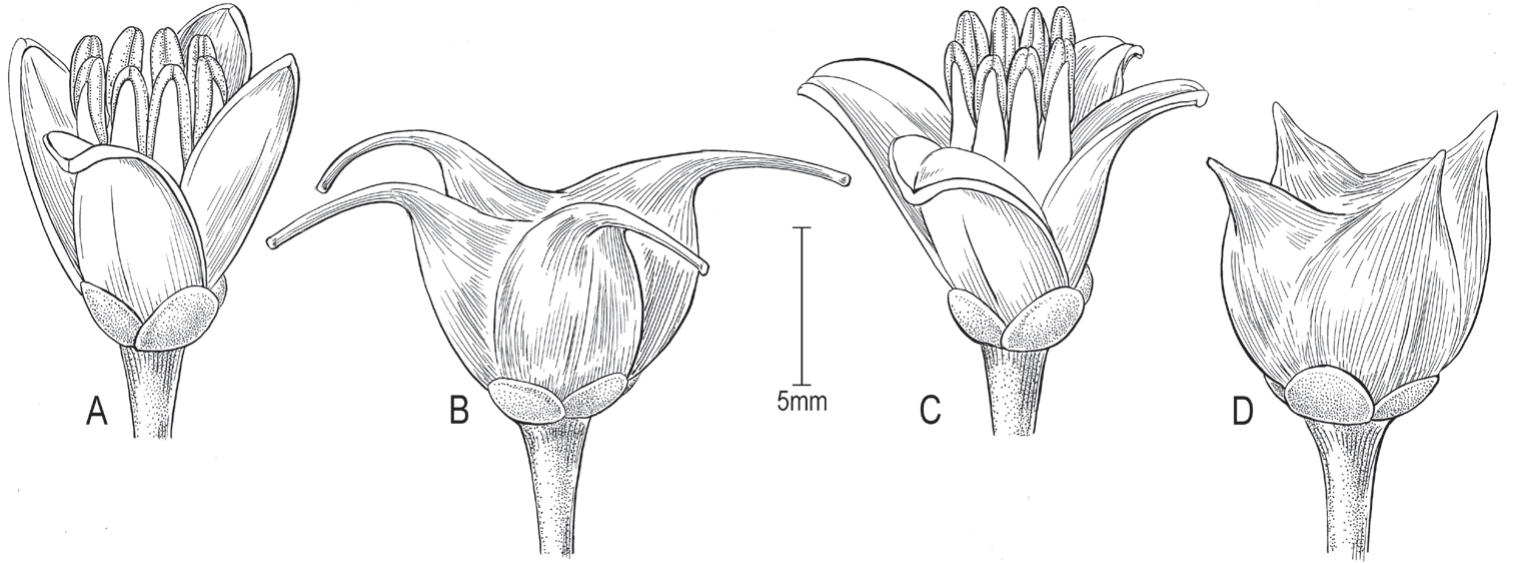
**A, B** *Melicope rostrata*, flower (field images of *Appelhans et al. MA683*, BISH, GOET), fruit (*Wood and DeMotta 14490*, US, and *Flynn 4626*, US) **C** Melicope cornuta var. decurrens flower (field images by Sebastian Marquez and Dave Fahrenwald in the Wai‘anae Mountains) **D** Melicope cornuta var. cornuta fruit (field images by Joel Lau in Nui, Ko‘olau Mountains).

### Melicope cornuta var. decurrens

Taxon classificationPlantaeSapindalesRutaceae

(B.C. Stone) Appelhans, K.R. Wood & W.L. Wagner comb. nov.

urn:lsid:ipni.org:names:77174270-1

Fig. 3C

Platydesma cornuta Hillebr. var. decurrens B.C. Stone, *J. Arnold Arbor*. 43: 423. 1962.

#### Type.

O‘ahu: Wai‘anae Mountains, Munia-Kanehoa trail, wet fern-covered banks by stream in valley just southeast of trail, [200 m], 26 March 1960, *B.C. Stone & G. Pearsall 3263* (holotype: BISH-579783 [BISH1016373, specimen!]).

### Melicope remyi

Taxon classificationPlantaeSapindalesRutaceae

(Sherff) Appelhans, K.R. Wood & W.L. Wagner comb. nov.

urn:lsid:ipni.org:names:77174271-1

Claoxylon remyi Sherff, *Publ. Field Mus. Nat. Hist., Bot. Ser*. 17: 557. 1939. Platydesma remyi (Sherff) O. Deg., I. Deg., Sherff & B.C. Stone, *Fl. Hawaiiensis*. 6: Fam. 179. 1960.
Platydesma campanulatum H. Mann var. sessilifolia Rock, *Indig. Trees Haw. Isl*. 243. 1913. – Type: Hawai‘i: in dense forests of the summit mountain of the Kohala range, 12 July 1909, *J. F. Rock 4222* (holotype: BISH, not located; isotype: GH [mounted on two sheets, GH00044160, GH00044161, images!]).

#### Note.

These two sheets are the only type material of this collection located. They were labelled as co-type, which was used at the time Rock published for the equivalent of isotype. Rock stated in his book that most of the specimens mentioned in the text were in the “college of Hawaii Herbarium”, which are now incorporated into BISH. He specifically indicated the type of this new variety to be there, so the presumed holotype should be at BISH, but could not be located.

#### Type.

Hawai‘i: 1851–1855, *J. Remy 604*, (holotype: P [P00636836, image!]; isotype: P [P00636837, image!]).

### Melicope rostrata

Taxon classificationPlantaeSapindalesRutaceae

(Hillebr.) Appelhans, K.R. Wood & W.L. Wagner comb. nov.

urn:lsid:ipni.org:names:77174272-1

Fig. 3A, B

Platydesma rostrata Hillebr., *Fl. Hawaiian Isl.* 72. 1888.

#### Note.

At the time Hillebrand described this species there was a single specimen known, the type. When Rock brought fragments from B back to BISH the label information was not well transcribed, but all have printed labels indicating they were from the B collection. There were no other collections in the B herbarium so despite the lack of information this must be a fragment of the holotype.

#### Type.

Kaua‘i: *V. Knudsen 68* (holotype: B-destroyed, fragment BISH-581794 [BISH1016395, specimen!]).

### Melicope spathulata

Taxon classificationPlantaeSapindalesRutaceae

A. Gray Bot. U.S. Expl. Exped. 1: 352. 1854. Platydesma spathulatum (A. Gray) B.C. Stone, Madroño. 16: 165. 1962.

Fig. 4

Melicope ? grandifolia A.Gray *Bot. U.S. Expl. Exped.* 1: 354. 1854. – Type: Hawai‘i: forests of Mauna Kea, 1840, *U.S. Expl. Exped. s.n.* (holotype: US-15033 [US00101457, specimen!]; isotype: GH [GH01153097, image!]).
Platydesma campanulata H. Mann, *Proc. Bost. Soc. Nat. Hist*. 10: 317. 1866. – Type: O‘ahu: on the mountains behind Honolulu, at middle heights, *H. Mann & W. T. Brigham 94* (holotype: CU, not located; isotypes: BISH [4 sheets, BISH1016385, BISH1016386, BISH1016388, BISH1016390, specimens!], G [G00380101, image!], GH [2 sheets, GH00044158, GH00044159, images!], MASS [MASS00320396, image!], MO [MO-251520, specimen!], NY [NY00067067, specimen!], US [US00101498, specimen!]).
Platydesma campanulata f. coriaceum Rock, *Indig. Trees Haw. Isl.* 243. 1913. – Type: Hawai‘i: Kohala Mts., [W of] Honokanenui gorge, June 1910, *J. F. Rock 8367* (holotype: BISH-586927 [BISH1016394, specimen!]; isotypes: BISH [BISH1016441, specimen!], GH [2 sheets, GH00044162, GH00044163, images!]).
Platydesma campanulata var. macrophylla Hillebr., *Fl. Hawaiian Isl*. 71. 1888. – Type: Kaua‘i: *V. Knudsen s.n.* (holotype: B-probably destroyed, fragment of holotype BISH-581799 [BISH1016383, specimen!]).
Platydesma campanulata var. pallida Hillebr., *Fl. Hawaiian Isl*. 71. 1888. Platydesma spathulatum var. pallidum (Hillebr.) B.C. Stone, *Madroño*. 16: 165. 1962. – Type: O‘ahu: Ka‘ala, *Hillebrand s.n*.; E. Maui, Hamakua, *Lydgate s.n.* (syntypes: B-destroyed); Maui: along pipe-line trail, Olinda, in dark forest, 29 July 1927, *O. Degener & L. Topping 8615* (neotype [designated by Stone, *J. Arnold Arbor.* 43: 420. 1962]: BISH-68071 [BISH1016379, specimen!]; isoneotypes: B [B_10_0296003, image!], BH [BH000121710, image!], K [K000342164, image!], MASS [MASS00320397, image!], NY [NY02859241]).
Platydesma campanulata var. pubescens Skottsb., *Acta Horti Gothob*. 15: 388. 1944. Platydesma spathulatum var. pubescens (Skottsb.) B.C. Stone, *Madroño*. 16: 165. 1962. – Type: O‘ahu: Wai‘anae Mountains, slope of Ka‘ala, 25 September 1938, *O. Selling 3710* (holotype: GB [GB-0048636, image!]; isotype: S [S08-7736, image!]).
Platydesma oahuensis H. Lév., *Repert. Spec. Nov. Regni Veg*. 10: 154. 1911. – Type: O‘ahu: Punalu‘u, May 1910, *U. Faurie 243* (holotype: not located; isotypes: A [A00044165, image!], BM [BM000798124, image!]), P [P00639232, image!]).
Platydesma spathulatum f. kalalauense O. Deg. & I. Deg., *Fl. Hawaiiensis*. 7: Fam. 179. 1960. – Type: Kaua‘i: east rim of Kalalau Valley, 16 Nov 1960, *O. Degener, I. Degener, & W. Cadenhead 27150* (lectotype [designated here]: US-2604492 [US00101496, specimen!]).
Platydesma spathulatum f. stonei O. Deg. & I. Deg., *Fl. Hawaiiensis*. 7: Fam. 179. 1960. – Type: O‘ahu: Ko‘olau range. Punalu‘u, summit of Castle trail, *B.C. Stone 3551* (holotype: BISH, not located; lectotype [designated here]: fig. 2, Stone, *J. Arnold Arbor.* 43: 418. 1962).

#### Type.

Kaua‘i: mountains, 1840, *U.S. Expl. Exped. s.n.* (holotype: US-15031 [US00101445, specimen!]).

**Figure 4.**
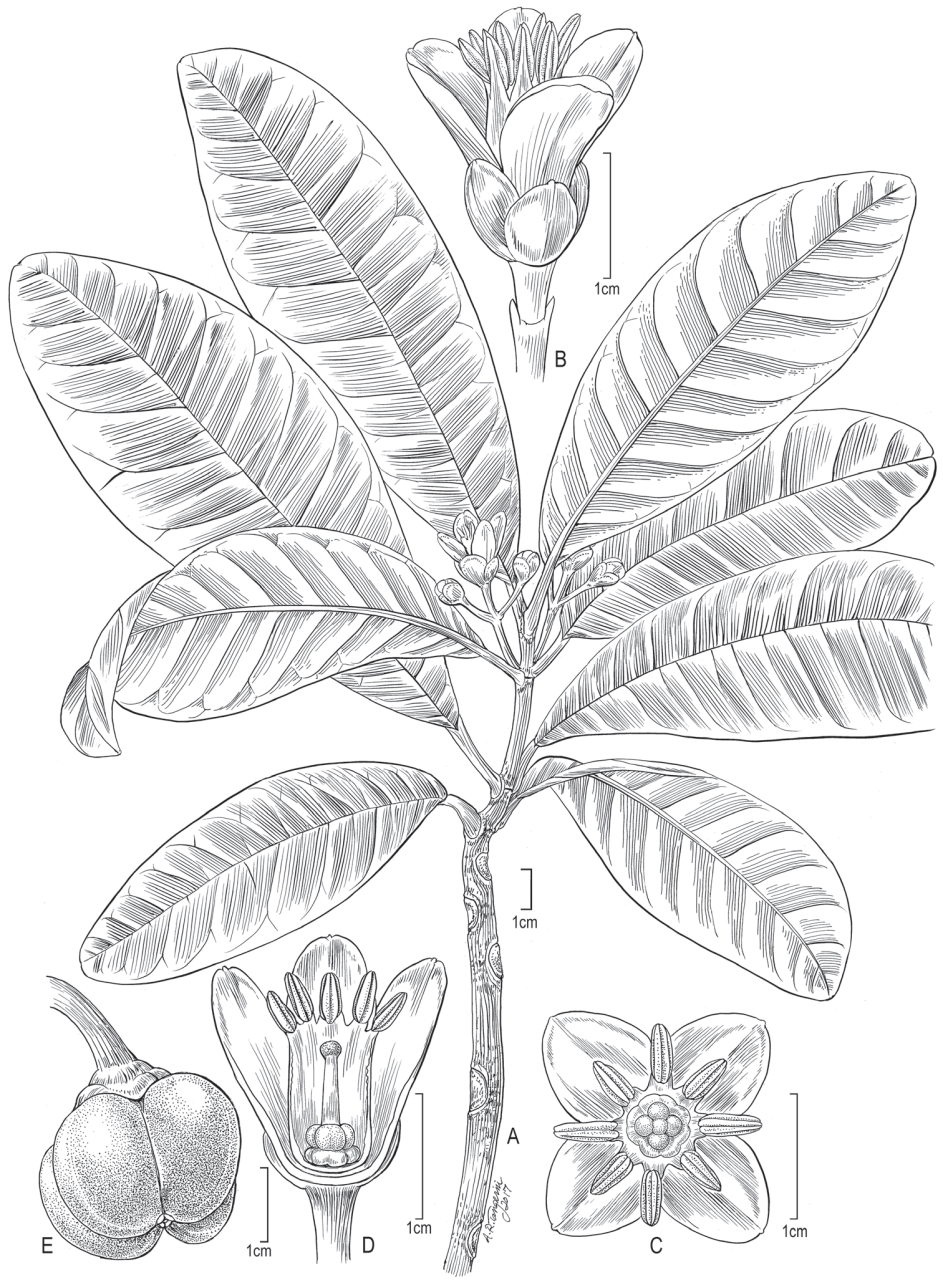
*Melicope spathulata* **A** habit with flower (*Wood 15091*, Kaua‘i, Kohua Ridge, US) **B** flower, lateral view (*Ishikawa 302*, Kaua‘i, Koke‘e, US) **C** flower, top view (*Ishikawa 302*, US) **D** flower, longitudinal section (*Ishikawa 302*, Kaua‘i, Koke‘e, US) **E** fruit (*Herbst & Mull 5507*, Hawai‘i, Ola‘a, US and *Takeuchi et al. 1997*, O‘ahu, Ko‘olau Mountains, US).

##### Names excluded from *Platydesma*

*Platydesma auriculifolia* (A. Gray) Hillebr., *Fl. Hawaiian Isl.* 72. 1888. *Pelea auriculifolia* A. Gray, *Proc. Amer. Acad. Arts* 3: 50. 1853. – Type: Hawai‘i: forests of Mauna Kea, *U.S. Expl. Exped., s.n.* (holotype: US-15020 [US00101488, specimen!]. [= *Melicope clusiifolia* (A. Gray) T.G. Hartley & B.C. Stone]. – Note: Hillebrand cited the second source of the name in *Pelea* by Gray (*U.S. Expl. Exped.*, Phan. 343. 1854) and also used part of the description (fruit). Hillebrand cites among the three collections the type of *Pelea auriculifolia* A. Gray as well as a specimen of *Platydesma* (*Hillebrand s.n.*), and the third one can’t be located (*Lydgate s.n.*). So his cited collections are a mixture as is the description. This Hillebrand name must be taken as a new combination. The critical facts are: Hillebrand cited the basionym, used the same epithet, and did not exclude the holotype of the basionym. It does not matter that Hillebrand cited a later publication of the cited basionym. Since this is a pre-1953 publication, it is not mandatory to cite the original publication of the basionym.

*Platydesma fauriei* H. Lév., *Repert. Spec. Nov. Regni Veg*. 10: 153. 1911. – Type: O‘ahu: Punaliuu [Punalu‘u], May 1910, *U. Faurie 242* (Isotypes: BM [BM000994065, image!], P [P00311275, image!]. – Note: The holotype was expected to be stored at E but no specimen could be located there. [=*Nothocestrum longifolium* A. Gray].

##### Insert for existing keys to Hawaiian Rutaceae and *Melicope*

The identification keys included in the “Manual of the Flowering Plants of Hawaii” (Wagner et al. 1990) need to be slightly modified in order to incorporate the taxonomic changes proposed here.

**Key to the genera of Rutaceae (p. 1175 in Wagner et al. 1990)**

**Table:** 

1	Leaves simple, opposite or whorled	**2. *Melicope***
–	Leaves pinnately compound, opposite, or alternate (2).
2	Leaves alternate; seeds not winged	**3. *Zanthoxylum***
–	Leaves usually opposite; seeds winged	**1. *Flindersia***

**Key to the species of Pelea/Melicope (p. 1178 in Wagner et al. 1990)**

**Table:** 

1	Shrubs, shrubby trees, or with palmoid habit; flowers perfect; petals slightly imbricate; filaments nearly completely connate into a staminal tube; ovules 5–8 per carpel; fruit a subglobose or cruciate capsule, indehiscent or tardily dehiscent	**use original key for *Platydesma*** (p. 1209 in Wagner et al. 1990)
–	Trees, shrubs, or subscandent; flowers functionally unisexual (and the plants polygamous) or rarely perfect; petals valvate; stamens in 2 whorls, distinct, reduced but always present in functionally pistillate flowers, longer fertile stamens equal to or exserted from corolla; ovules 2 per carpel; fruit composed of 4 nearly distinct follicles or a 4-lobed, 4-valved, cruciate or cuboid capsule, dehiscent	**use original key to species of *Pelea/Melicope*** (p. 1178 in Wagner et al. 1990)

## Supplementary Material

XML Treatment for Melicope sect. Pelea

XML Treatment for Melicope cornuta

XML Treatment for Melicope cornuta var. decurrens

XML Treatment for Melicope remyi

XML Treatment for Melicope rostrata

XML Treatment for Melicope spathulata
